# Screening of Microalgal Species for Biostimulant and Biofertilizer Applications

**DOI:** 10.3390/md24070228

**Published:** 2026-06-29

**Authors:** Eirini Sventzouri, Eleni Pagkaki, Sotirios Zerveas, Giorgos Markou, Michael Kornaros

**Affiliations:** 1 Laboratory of Biochemical Engineering & Environmental Technology (LBEET), Department of Chemical Engineering, University of Patras, 26504 Patras, Greece; eirinisventzouri@gmail.com (E.S.); helenpag@hotmail.com (E.P.); 2 Institute of Technology of Agricultural Products, Hellenic Agricultural Organization—DIMITRA, Sof. Venizelou 1, 14123 Lykovrysi, Greece; sotzerveas@gmail.com (S.Z.); markougior@elgo.gr (G.M.)

**Keywords:** microalgae, agriculture, biofertilizers, biostimulants, environmental sustainability

## Abstract

Microalgae represent a promising alternative as biofertilizers and biostimulants, providing essential nutrients and bioactive compounds that support plant growth. In this study, a screening of seven microalgal species—including *Arthrospira platensis*, *Nannochloris* sp., *Chlorella* sp., *Chlorella vulgaris*, *Acutodesmus obliquus*, *Parachlorella kessleri*, *Coelastrella vacuolata*—and one isolated mixed culture was conducted to evaluate their potential as biostimulants and biofertilizers under autotrophic cultivation conditions. Whole cultures and corresponding supernatants were directly applied, without any pretreatment, reducing potential processing costs. Their biostimulant activity was evaluated through multiple bioassays, including germination index and auxin- and cytokinin-like responses, while nitrogen, phosphorus, and potassium content was analyzed to assess biofertilizer potential. The results revealed that biostimulant effects were strongly influenced by species, concentration, and sample fraction. *Chlorella* species consistently showed high performance across assays, combining strong germination and rooting responses with high nitrogen content (8.2–8.8% *w*/*w*), while *A. platensis* and *Nannochloris* sp. showed inhibitory effects in many cases. Overall, under the cultivation and application conditions tested, *C. vulgaris*, mixed culture, and *A. obliquus* are identified as promising candidates for combined biostimulant and biofertilizer applications. This study is a primary step in identifying the most promising species as an alternative to synthetic fertilizers, enabling further optimization towards more sustainable agricultural practices.

## 1. Introduction

The global population has been steadily increasing, and it is expected to reach nine billion by 2050 [1,2]. This growth is expected to significantly increase the demand for food, placing considerable pressure on agricultural systems and requiring enhanced productivity to meet future needs [3]. In addition to increasing food demands, agriculture has also to cope with resource depletion and climate change impacts [4]. Indeed, the excessive use of chemical fertilizers has led to several environmental and agronomic issues, including imbalances in nitrogen, phosphorus, and potassium ratios, soil degradation, nutrient depletion, and groundwater contamination, while these toxic compounds in soil and crops pose risks to both human health and the environment [3,5]. For example, 80% of the nitrogen produced worldwide is used in inorganic fertilizers, which are generated through energy-intensive processes, and it is estimated that around 40% of the applied nitrogen is subsequently lost to the environment [6]. Consequently, reducing dependence on chemical fertilizers and identifying sustainable and environmentally friendly alternatives has become a key priority.

In this context, the production of biostimulants and biofertilizers production is a promising technique to bring greater sustainability in agricultural development and address the challenges associated with chemical fertilizers [7,8]. Biofertilizers consist of living microorganisms or natural substances that improve soil fertility and nutrient availability, thereby supporting plant growth. Biostimulants, on the other hand, are organic-derived products that enhance plant development by stimulating physiological processes [9]. Among the different production methods, microalgae and cyanobacteria have attracted increasing attention as a valuable resource for crop production and protection due to their combined biofertilizing and biostimulating properties [10,11]. These photosynthetic microorganisms contain valuable compounds, such as amino acids, carbohydrates, minerals, trace elements, and phytohormones such as auxins, gibberellins, and cytokinins that can enhance plant growth [12,13]. The application of microalgal biomass to soil has been shown to improve its physicochemical properties, enhance enzymatic and microbial activity, and provide a slow-release source of organic nutrients [14]. Moreover, microalgae and cyanobacteria can be produced on non-arable land, while cyanobacteria are capable of fixing approximately 25 Gt of carbon annually into energy-dense biomass using atmospheric CO_2_ and solar energy [2,12].

Despite the growing interest and the environmental benefits of these microalgae-derived bioproducts, only a limited number of well-characterized and consistently stable products are currently available on the market as they cannot yet compete with the price of chemically based ones [3]. Scaling up such systems for industrial deployment presents several challenges, including the economic viability of the process [15]. Cultivation in wastewater [14,16,17] and the implementation of a biorefinery concept, with simultaneous extraction of other value-added products, can enhance the economic feasibility of the process [14,18]. Downstream processes such as the extraction of bioactive compounds significantly increase operational costs. Consequently, selecting specific algal strains or optimizing growth conditions becomes a strategic priority to avoid complex downstream steps, favoring instead the use of whole wet biomass or easily recoverable extracellular products [19]. Downstream extraction of intracellular bioactive compounds can substantially increase processing costs, and the high energy demand of downstream processing is a major barrier to the commercial sustainability of microalgae-based biostimulants. Therefore, selecting strains that are effective as whole cultures, dry biomass, spent medium, or cell suspensions, or that release active compounds without intensive extraction, is a practical strategy to reduce processing requirements and improve economic feasibility [20].

Different microalgal species exhibit distinct bioactivity profiles. While some act as growth biostimulators, others may induce inhibitory responses depending on their biological composition [21]. Moreover, parameters such as concentration and the form of application, including whole cultures or cell-free extracts, can significantly influence the observed biological effects [22]. The effectiveness of microalgal products is often associated with their dual functionality, combining nutrient supply with biostimulant activity, although their performance ultimately depends on species, composition, and application conditions [23]. These variations highlight the complexity of microalgal-based systems and underline the need for systematic screening approaches. However, practical application also requires consideration of safety and regulatory issues, as not all microalgal species are suitable for food, feed, or environmental use. Only a limited number have obtained Generally Recognized as Safe (GRAS) status or equivalent safety recognition, mainly because this category requires costly and time-consuming safety testing [24]. Therefore, strain-specific safety assessment and compliance with the relevant regulatory framework remain essential before commercial application and must be carefully considered during the early stages of product development.

The scope of the present study was the evaluation of seven microalgal species, including *Arthrospira platensis*, *Nannochloris* sp., *Chlorella* sp., *Chlorella vulgaris*, *Acutodesmus obliquus*, *Parachlorella kessleri*, *Coelastrella vacuolata*, and an isolated mixed culture, in terms of their potential use as biostimulants and biofertilizers. In addition to monocultures, mixed microalgal and cyanobacterial cultures or consortia have attracted interest for agricultural applications because different microorganisms may provide complementary functions. Previous studies have reported that consortium-based systems can lead to yield improvement, reduced fertilizer dependency, enhanced nutrient-use efficiency, improved plant physiological performance, better crop quality, and potential soil fertility enhancement [25,26], although their agronomic potential has been less extensively studied than that of individual strains. Whole cultures and corresponding supernatants, produced under autotrophic cultivation conditions, were applied without any pretreatment in different bioassays including germination index and auxin- and cytokinin-like responses. Nutrient composition of the produced biomass was also evaluated to assess its potential as biofertilizer. Although some of these species have been evaluated individually under specific conditions, comparative screening of untreated fractions, including whole cultures and cell-free supernatants, across different hormone-like bioassays remains limited. This represents an important gap, because the direct use of crude cultures or supernatants could reduce the need for energy-intensive downstream processing, but their biological performance is expected to depend strongly on species, concentration, and application fraction. Specifically, the objectives of this study were: (i) to compare the effects of whole cultures and corresponding supernatants on bioassay tests, (ii) to determine whether these responses are species-, concentration-, and fraction-dependent, and (iii) to integrate bioactivity data with nutrient biomass composition in order to prioritize candidate strains for subsequent agronomic validation. This research aims to provide an integrated evaluation of microalgal species and identify the most promising candidates for sustainable agricultural applications.

The species evaluated in this study include a variety of photosynthetic microorganisms with diverse ecological origins and applications. *Arthrospira platensis* is a photosynthetic, multicellular, and filamentous cyanobacterium, which is widely used as a food supplement due to its high content of proteins, unsaturated fatty acids, vitamins, and minerals [27,28]. *A. platensis* can adapt to a wide range of aquatic environments such as fresh, brackish, or seawater. Previous studies have demonstrated the adaptability of *A. platensis* to saline conditions, with successful long-term cultivation reported at salinities ranging from 5 to 60 g L^−1^ NaCl using natural sea salt [28]. *Nannochloris* sp. is considered a promising species because of its high tolerance to elevated bicarbonate levels and salinity, as well as its potential applications in food, feed, and carbon capture technologies [29]. *Chlorella* strains, *Parachlorella kessleri*, *Acutodesmus obliquus*, and *Coelastrella vacuolata* are predominantly freshwater unicellular microalgae with diverse biotechnological applications, including food and feed production, biodiesel production, and wastewater bioremediation. Nevertheless, several studies have demonstrated their ability to tolerate and adapt to saline conditions, indicating their potential cultivation under marine or brackish environments [30,31,32,33]. For example, a *C. vulgaris* strain isolated from a freshwater environment in New Zealand exhibited similar growth rates in media prepared with freshwater and 50% seawater, while reduced growth was observed under 100% seawater conditions, with oil productivity being doubled compared to freshwater. *P. kessleri* achieved higher productivity in the brackish effluent compared to the synthetic control medium, suggesting the effluent provides a more robust nutritional matrix [31]. Overall, the inclusion of species with different environmental adaptations broadens the evaluation of their potential for sustainable agricultural applications.

## 2. Results and Discussion

### 2.1. Evaluation of Biostimulant Potential

The biostimulant potential of the eight selected microalgal cultures was evaluated using different bioassay tests. Germination index (GI), mung bean rooting, and cucumber bioassays were used to evaluate gibberellin-, auxin- and cytokinin-like responses, respectively, according to previously established methodologies [4,13,34,35]. These bioassays were used as functional screening tools and do not directly confirm the presence or concentration of specific phytohormones. Both whole cultures and supernatants were applied at different concentrations to investigate their effects.

#### 2.1.1. Gibberellin-like Effect: Germination Index

**Figure 1:**
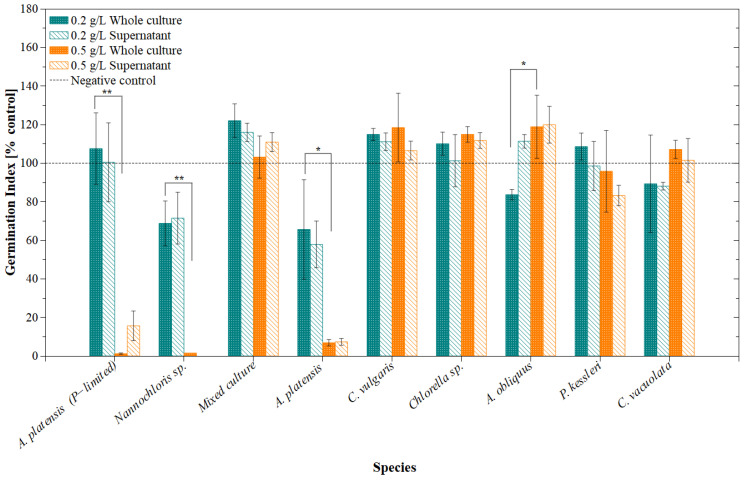
Germination index (%) of cucumber seeds treated with whole cultures and supernatants of different microalgal species at 0.2 and 0.5 g L^−1^, considering deionized water as the negative control (100%). Error bars represent the standard deviation (*n* = 3). Asterisks indicate statistically significant differences between the two whole culture concentrations (0.2 and 0.5 g L^−1^) within each species. Pairwise comparisons are indicated by brackets, * *p* < 0.05; ** *p* < 0.01.

Figure 1 presents the effects of whole cultures and supernatants on the GI of cucumber seeds at concentrations of 0.2 and 0.5 g L^−1^. The results are presented as percentages relative to the negative control. Most of the treatments’ GI values were close to or exceeded 100%, demonstrating biostimulant activity. In general, a GI value below 50% indicates high phytotoxicity, while values between 50% and 80% reflect moderate inhibitory effects. In contrast, GI values above 80% are considered non-toxic, and values equal to or exceeding 100% indicate a stimulatory effect on seed germination and early plant growth [36]. A three-way ANOVA (Table S1) revealed that species, concentration, and sample fraction, as well as their interactions, significantly affected GI (*p* < 0.05), confirming that the response depends on the specific combination of factors. Among the main effects, concentration showed the highest contribution to the observed variation (48.92%), followed by species (27.31%), whereas the contribution of sample fraction was lower (3.71%). Species-level analysis revealed that species including *Chlorella* strains and mixed culture exhibited high GI values (>100%), with no statistically significant effects of concentration or sample fraction (*p* > 0.05), indicating a stable biostimulant response. In contrast, both whole culture and supernatant of *A. platensis*, regardless of cultivation condition, and *Nannochloris* sp. at 0.5 g L^−1^ exhibited a significant phytotoxicity compared to 0.2 g L^−1^ (*p* < 0.05). Decrease in GI value with increasing applied concentration has also been reported in the literature for several species [4,20,37,38,39], suggesting that lower concentrations might be necessary to avoid toxicity. Phytohormones are bioactive compounds that influence physiological processes at low concentrations but can inhibit at higher concentrations [37]. Other biologically active compounds, including phenols, fatty acids and polysaccharides, are also usually present. At higher concentrations, some of these compounds may have an inhibitory effect that counteracts the positive, promoting effects of phytohormones [20]. However, because these compounds were not chemically characterized in the present study, the mechanisms underlying the observed inhibition cannot be verified. Post hoc analysis (Tukey test) supported these findings, with *Chlorella* species and the mixed culture consistently classified within the highest statistical groups, whereas *A. platensis* and *Nannochloris* sp. at 0.5 g L^−1^ were assigned to the lowest group, confirming their inhibitory effect (Table S2). *Chlorella* strains have been reported to enhance germination index, either through direct application [40,41] or following biomass pretreatment [39,42,43]. In the study of Viegas et al. [40], application of *C. vulgaris* and *T. obliquus* cultures at 0.2 g L^−1^ to wheat seeds caused a 147% and 101% increase in GI compared to control, respectively. Ferreira et al. [13] investigated the GI of *C. vulgaris* in different crops at a concentration of 0.5 g L^−1^. The highest GI value, almost 180%, was reported for cucumber seeds, while for the other seeds values ranged between 100% and 150%. These effects can be attributed to bioactive molecules such as fatty acids, polysaccharides and phytohormones [13] and especially gibberellin-like compounds, which have been reported to play a critical role in initiating seed germination [12,20]. In the present study, however, no direct chemical analysis was conducted, and the contribution of these compounds remains hypothetical. Overall, the bioassay revealed that several of the tested treatments had a positive effect on seed germination indicating the presence of bioactive compounds with potential biostimulant application.

#### 2.1.2. Auxin-like Effect: Mung Bean Rooting Bioassay

**Figure 2:**
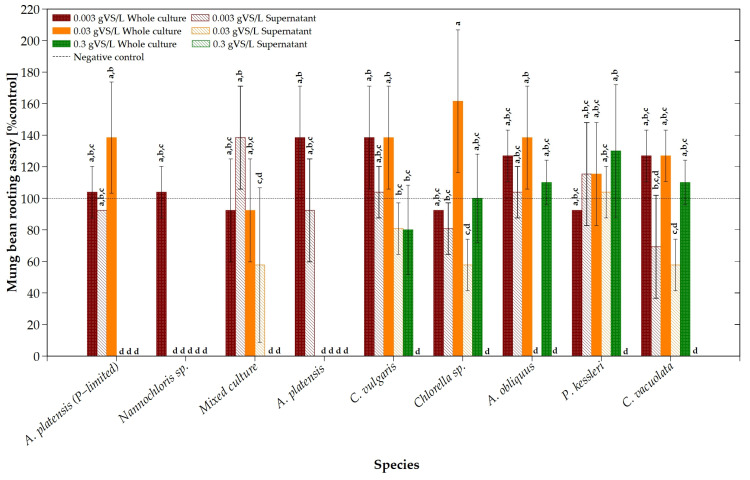
Mung bean rooting assay (%) treated with whole cultures and supernatants of different microalgal species at 0.003, 0.03 and 0.3 gVS L^−1^, considering deionized water as the negative control (100%). Error bars represent the standard deviation (n = 3). ^a,b,c,d^ Data that do not share a letter are significantly different based on Tukey test, *p* < 0.05.

The auxin-like effect of the examined microalgal cultures was assessed using the mung bean rooting bioassay (Figure 2). Auxins, such as indole-3-acetic acid and indol-3-butyric acid, have been found to be present in microalgae and play a crucial role in the induction of root formation [12,44]. A three-way ANOVA (Table S3) indicated that species, concentration, fraction and their interactions significantly affected the root formation (*p* < 0.001), demonstrating that effects are influenced by the specific combination of experimental conditions. Among the main effects, fraction showed the highest contribution to the observed variation (48.93%), followed by concentration (35.77%), whereas species had a lower contribution (6.12%). This indicates that the form in which the microalgal material was applied, was the major determinant of the rooting response. The significant interaction terms further suggest that the effect of concentration depended on both the species and the applied fraction, supporting the need for treatment-specific interpretation rather than conclusions based only on single factors. Tukey’s post hoc test further confirmed the differentiation among treatments, separating the highest rooting responses from the inhibitory treatments (Figure 2). Treatments that did not share common letters were significantly different (*p* < 0.05). Whole culture treatments, particularly those of *Chlorella* sp., *C. vulgaris*, *A. obliquus* and *P. kessleri*, were generally assigned to the upper statistical groups, whereas several supernatant treatments, especially at 0.3 gVS L^−1^, were classified in the lowest group, suggesting their inhibitory effect on root formation. Overall, several treatments, particularly whole cultures at 0.03 gVS L^−1^, exhibited values above the negative control, indicating a potential stimulatory effect. Specifically, *Chlorella* sp., *C. vulgaris*, and *A. obliquus*, achieved rooting formation values of 160%, 140%, and 140%, respectively. *A. obliquus* has been reported to contain endogenous phytohormones, including abscisic acid (ABA) and the auxin indole-3-acetic acid (IAA), at concentrations of approximately 42 and 200 amol cell^−1^, respectively [45]. Similar results were obtained in the study of Mutum et al. [38], when *C. vulgaris* biomass achieved a mung bean rooting formation equal to 150% at a concentration of 1 g L^−1^. In general, whole cultures consistently outperformed their respective supernatants, which frequently exhibited reduced or inhibitory effects, in promoting root formation, indicating that the responsible bioactive compounds were probably intracellular and gradually released from the cells during the bioassay. During the fifteen-day bioassay, whole cells may have progressively lysed providing a slow release of biostimulants. Conversely, since the supernatants were applied without any pretreatment, extraction, or cell disruption step, they contained only the compounds that had already been released into the culture medium prior to application. Therefore, they may have lacked part of the intracellular or biomass-associated bioactive compounds.

In the study of Ferreira et al. [4], supernatant application of *T. obliquus* after the enzymatic hydrolysis of the cells significantly enhanced the percentage of rooting (285%) compared to the supernatant from untreated cells, which was below that of the negative control. However, the efficacy of cell disruption is highly dependent on the method used. For instance, Stirk et al. [46] observed that intensive mechanical methods like bead-milling or sonication reduced rooting in mung beans. They attributed this to the potential degradation of sensitive biostimulants or the simultaneous release of inhibitory compounds during the extraction process. In addition, a concentration-dependent response was evident. While moderate concentrations (0.03 gVS L^−1^) tended to promote higher rooting values, increasing the concentration to 0.3 gVS L^−1^ resulted in a significant decline in activity. This suggests that low to moderate concentrations are preferred, as high auxin concentration can inhibit growth [47]. Moreover, the total lack of root formation when the highest concentration of supernatants was applied indicates a concentration-dependent inhibitory response and may also suggest the release of inhibitory and phytotoxic substances during growth. However, chemical characterization would be required to determine the underlying cause.

#### 2.1.3. Cytokinin and Auxin-like Effect: Cucumber Cotyledon Bioassays

**Figure 3:**
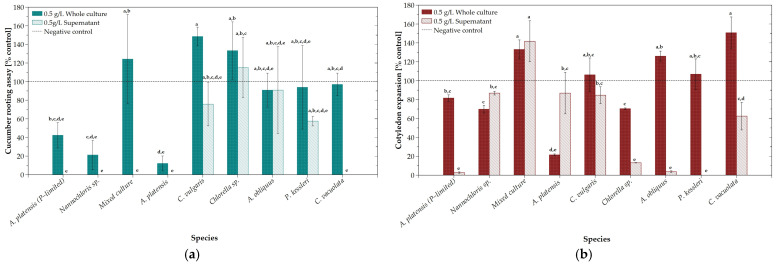
Cucumber cotyledon bioassays (%) treated with whole cultures and supernatants of different microalgal species at 0.5 g L^−1^, considering deionized water as the negative control (100%): (**a**) rooting bioassay (auxin-like effect) and (**b**) cotyledon expansion (cytokinin-like effect). Error bars represent the standard deviation (n = 2). ^a,b,c,d,e^ Data that do not share a letter are significantly different based on Tukey test, *p* < 0.05.

The auxin- and cytokinin-like effect of the microalgal species was further evaluated through the cucumber cotyledon bioassays. In the cucumber rooting bioassay, the mean value of only four samples exceeded the negative control, indicating potential biostimulator activity. Whole cultures of *C. vulgaris* and *Chlorella* sp. achieved the highest responses equal to 149% and 133%, respectively. The mixed culture also showed a stimulatory response in the cucumber rooting assay, but only when applied as whole culture, reaching approximately 124% of the control. In contrast, its supernatant caused complete inhibition of root formation. *Chlorella* strains were also in the highest group at mung bean rooting bioassay (Figure 2). A two-way ANOVA (Table S4) showed that both species and fraction significantly affected cucumber rooting and cotyledon expansion (*p* < 0.001). However, as these bioassays were performed in duplicate, the statistical results should be considered preliminary for screening purposes. For cucumber rooting, fraction was the dominant source of variation, accounting for 68.26% of the response variability, whereas species contributed 25.67%. This suggests that, under the tested conditions, the application form, whole culture or supernatant, may have had a stronger effect than the microalgal species itself. Tukey’s post hoc test further supported this pattern, as whole cultures were generally classified in higher statistical groups than the corresponding supernatants, several of which were assigned to the lowest group due to complete or near complete inhibition of root formation. The same trend was observed in both rooting bioassays regarding the supernatant application, which often led to complete inhibition of root formation. Differences in root formation between mung bean and cucumber bioassays have been reported in the literature even under identical treatment concentrations, indicating that auxin-like responses also depend on the plant species and the assay used [46]. Therefore, the evaluation of auxin-like activity cannot be considered assay-independent, and conclusions based on a single bioassay may be misleading. In the cotyledon expansion test (Figure 3b), some treatments exhibited values exceeding the negative control indicating a possible cytokinin-like effect. The whole culture of *C. vacuolata* and the whole culture and the supernatant of the mixed culture reached values of 150%, 133% and 142%, respectively. Up to 17 cytokinin types have been detected in different *Chlorella* strains, with total cytokinin contents ranging from 3.3 to 7.1 nmol g^−1^ dry weight [48]. Interestingly, the mixed culture showed a strong cytokinin-like response in both fractions, with the supernatant exhibiting slightly higher activity than the whole culture. This suggests that, in this case, the compounds responsible for cotyledon expansion were at least partly extracellular or readily released into the culture medium. In a previous study, Ferreira et al. [4] tested whole cultures and the corresponding supernatants of *T. obliquus*, with and without pretreatment, at the cotyledon expansion. Despite the observed auxin-like effects at both mung bean and cucumber rooting, almost all treatments did not show a cytokinin-like effect. In the cotyledon expansion assay, supernatants also exhibited biological activity, although often close to or below the negative control. However, in some cases, higher responses were observed, indicating the release of bioactive compound(s). The inconsistency in auxin- and cytokinin-like responses across species suggests that the optimal concentrations of these two phytohormones may differ. Concentrations that promote auxin-like activity may not be optimal, or may even be inhibitory, for cytokinin-like responses. In the study of [44], different extracts of *S. obliquus* were applied in cucumber bioassays at concentrations of 0.5 and 2 g L^−1^. Although root formation did not differ between the two concentrations, a significant positive effect was observed in cucumber expansion at 2 g L^−1^. This suggests that lower amounts of auxin may be accumulated, requiring higher concentrations to detect their positive effects. Notably, the species with the highest mean values in cucumber rooting were not the same as those showing the highest cytokinin-like activity. This observation may be further explained by the antagonistic roles of auxins and cytokinins in plant development, where auxins promote root initiation while cytokinins can inhibit it, potentially leading to differential responses depending on the dominant bioactive compounds produced by each species [49].

### 2.2. Biofertilizer Potential

Table 1 presents the nitrogen, phosphorus, and potassium content of the produced microalgal biomasses. Nitrogen content ranged between 4.3% and 8.8% *w*/*w*, with the highest values reported for *Chlorella* sp., *A. obliquus*, and *C. vulgaris* equal to 8.8%, 8.4%, and 8.2% *w*/*w*, respectively. *A. platensis* cultivated under P-limited conditions exhibited significantly lower nitrogen content (4.3% *w*/*w*), reflecting the impact of nutrient limitation on biomass composition. In general, cultivation conditions significantly influence biomass nutrient content and composition. For example, under nitrogen-rich cultivation conditions, microalgae tend to accumulate high levels of protein in their biomass, as proteins constitute the main nitrogen-containing cellular components [50]. Interestingly, mixed culture exhibited high phosphorus content (4.6% *w*/*w*), followed by *C. vulgaris* (3% *w*/*w*). Previous studies report improved phosphorus removal and increased phosphorus availability in soil and plant tissue involving mixed microalgal cultures and cyanobacteria consortia, respectively [26,51]. In the study of Slinksienė et al. [52], optimized cultivation of *Chlorella* sp. in landfill leachate resulted in phosphorus content equal to 2.1%. Potassium content was generally lower across all species, ranging from 0.0% to 2.1% *w*/*w*, with the highest values observed in *A. platensis* (2.1% *w*/*w*) and *C. vulgaris* (1.3% *w*/*w*). Typically, potassium content in microalgae ranges between 0.5% and 2% of dry weight, depending on the species and cultivation conditions [50]. Overall, the results showed that most of the species tested had a high nutrient content, which was much higher than that of other types of organic fertilizers, such as cow and pig manure [53].

Microalgal biomass rich in nitrogen and phosphorus can be used as biofertilizer, gradually providing the nutrients to plants while reducing the risk of nutrient losses associated with conventional fertilizers. The application of microalgal biomass as a biofertilizer represents one of its most common and effective uses, enhancing plant growth primarily through improved nutrient availability in the rhizosphere [9,54]. Several species have been successfully used as biofertilizers. *C. vulgaris* and *A. platensis* dried biomass has been used as a biofertilizer for rice and maize crops, improving the availability of macronutrients and increasing plant yields [55,56]. Table 2 summarizes examples of different microalgae species applications in agriculture and their potential use as biofertilizers. The nutrient composition obtained in the present study is broadly comparable with the values reported in Table 2 for microalgal biomass previously evaluated as biofertilizers. In particular, the nitrogen content of the tested cultures, ranging from 4.3 to 8.8% *w*/*w*, is similar to or higher than several literature examples, while phosphorus and potassium contents fall within comparable ranges.

### 2.3. Comparative Evaluation of Biostimulant and Biofertilizer Potential

The results indicate a clear potential for microalgal biomass as both biostimulant and biofertilizer. However, the observed responses were highly complex and strongly dependent on multiple interacting factors, including species, concentration, and sample fraction. In the present study, the cultures tested were not produced under fully standardized cultivation conditions. These differences may have influenced biomass composition and the production or release of bioactive compounds. Therefore, the differences observed among species should not be interpreted solely as intrinsic species-specific physiological responses and the present work should be considered as an initial comparative screening of cultures produced under the applied autotrophic cultivation conditions rather than as a strictly controlled comparison of species physiology. Statistical analysis revealed that the biostimulant response was strongly dependent on the concentration and the fraction applied, while species had a secondary but significant effect.

For the GI and cucumber bioassays concentration accounted for the largest proportion of variability equal to 48.9 and 68.3%, respectively (Tables S1 and S4). Sample fraction was the dominant factor in the mung bean rooting assay (48.9%), highlighting the importance of both dosage and the form of application (Table S3). Across bioassays, *Chlorella* species (*Chlorella* sp. and *C. vulgaris*) exhibited the most consistent performance, showing high germination index values, strong auxin-like activity, and moderate cytokinin-like responses, while *A. platensis* and *Nannochloris* sp. exhibited inhibitory effects under certain conditions, limiting their applicability. In the study of Charoenying et al. [62], *S. platensis* extracts and isolated compounds inhibited seed germination and seedling growth of Chinese amaranth and barnyard grass. The active fractions included fatty acids and norisoprenoids, with inhibition reported in the 50–1000 ppm range and complete inhibition in some treatments. However, different phytohormones such as IAA, PAA, and IBA have been detected in dry biomass of *A. platensis* at concentrations up to 8000, 195,400, and 15 ng g^−1^, indicating that the fraction applied should be carefully selected based on specific species [63]. The mixed culture also showed strong overall performance, particularly in germination index, cotyledon expansion, and phosphorus content. This suggests that mixed cultures may provide multifunctional effects through the combined contribution of different microorganisms. Nevertheless, since the present study did not investigate microbial composition, the possible role of synergistic interactions should be interpreted cautiously and further investigation is necessary. The literature indicates that the heterogeneous composition of mixed microalgal and cyanobacterial cultures can strongly influence the performance of biostimulant and biofertilizer products. Mixed cultures may improve efficacy by broadening the range of metabolites, nutrients, and signaling compounds available [64]. Experimental evidence from consortium-based systems shows that mixed cultures can perform well at low concentrations but become less effective or even inhibitory at higher concentrations [65]. In the present study, *Chlorella* species exhibited a comparatively balanced nutrient profile combined with consistent biostimulant activity, supporting their suitability as multifunctional biofertilizer candidates. *A. platensis*, despite its relatively high potassium levels, is constrained by its variable and occasionally inhibitory biostimulant responses.

To synthesize the diverse experimental responses, an exploratory comparative scoring approach was applied (Table 3). Min max normalization was selected because it rescales indicators with different units and ranges into a common interval between 0 and 1, allowing their combined comparison within a composite score. Equal weighting was then applied during aggregation. This approach was selected as a simple and transparent strategy, since no objective or experimentally validated basis was available to assign different weights to the individual bioassay responses and nutrient-content parameters. Equal weighting is commonly used in composite-indicator approaches when no objective basis for differential weighting is available; however, it remains a simplifying assumption [66]. Therefore, Table 3 should be interpreted as an exploratory prioritization tool rather than as a validated decision-making framework. Overall, this preliminary screening identified *C. vulgaris*, the mixed culture and *A. obliquus* as promising candidates for further validation for the production of microalgal biostimulants and biofertilizers due to their comparatively high combined performance.

## 3. Materials and Methods

### 3.1. Microalgal Species and Cultivation Conditions

A total of eight photosynthetic cultures were used in the present study. Specifically, the cyanobacterium *Arthrospira platensis* SAG 21.99 and the microalgae *Chlorella vulgaris* SAG 211-11b, *Acutodesmus obliquus* SAG 276-6, *Parachlorella kessleri* SAG 211-11g and *Coelastrella vacuolata* SAG 211-8b were obtained from SAG Culture Collection (University of Göttingen). *Nannochloris* sp. JB17 (isolated in house), *Chlorella* sp. (isolated from the natural environment in Algeria, Centre de Développement des Énergies Renouvelables) and a mixed culture (recovered from the liquid fraction of anaerobic digestion effluent originating from treated agro-industrial residues and wastewater) were also investigated. The mixed culture was a consortium of different microalgal and cyanobacterial species, which were initially formed through the inoculation of diluted digestate with *Chlorella vulgaris*, *Nannochloris* sp. JB17, *Nannochloropsis acculata* and *A. platensis* to form an acclimatized ecosystem. This mixed culture was maintained for several months with periodic renewal of the cultivation medium (modified BG-11 medium with 0.4 g L^−1^ K_2_HPO_4_). However, the exact consortium composition is not known, since it is expected that other microorganisms (bacteria, fungi, etc.) would be present.

Τo produce the required biomass quantity for the subsequent bioassays and compositional analyses, all species were cultivated autotrophically in batch mode and harvested at the end of their exponential growth phase, ensuring high cell viability. *C. vulgaris*, *Chlorella* sp., *A. obliquus*, *P. kessleri*, *C. vacuolata*, and mixed culture were cultivated in BG-11 medium (73816, Sigma-Aldrich, St. Louis, MO, USA) supplemented with a trace metal solution (Mix A5 with Co 92949, Sigma-Aldrich). *A. platensis* was cultivated in “Spirulina medium”, which contained the following (g L^−1^): NaHCO_3_ (13.61), Na_2_CO_3_ (4.03), K_2_HPO_4_ (0.50), NaNO_3_ (2.50), K_2_SO_4_ (1.00), NaCl (1.00), MgSO_4_·7H_2_O (0.20), CaCl_2_·2H_2_O (0.04), FeSO_4_·7H_2_O (0.01), EDTA (Titriplex III, Merck, Rahway, NJ, USA) (0.08), and 0.5% *v*/*v* micronutrient solution containing the following (g L^−1^): ZnSO_4_·7H_2_O (0.001), MnSO_4_·7H_2_O (0.002), H_3_BO_3_ (0.01), Co(NO_3_)_2_·6H_2_O (0.001), Na_2_MoO_4_·2H_2_O (0.001), CuSO_4_·5H_2_O (0.0005·10^−3^), FeSO_4_·7H_2_O (0.7), and EDTA (Titriplex III, Merck) (0.8). For phosphorus-limited conditions, Zarrouk medium which contained the following (g L^−1^) was used: NaHCO_3_ (16.8), NaNO_3_ (2.5), K_2_SO_4_ (1.0), NaCl (1.0), CaCl_2_ (0.04), Na_2_EDTA (0.08), MgSO_4_·7H_2_O (0.2), FeSO_4_·7H_2_O (0.01) and 1.0 mL of trace elements (g L^−1^): H_3_BO_3_ (2.86), (NH_4_)_6_Mo_7_O_24_ (0.02), MnCl_2_·4H_2_O (1.8), Cu_2_SO_4_ (0.08), and ZnSO_4_·7H_2_O (0.22). *Nannochloris* sp. was cultured in Zarrouk medium with increased NaHCO_3_ concentration (25 g L^−1^) [29]. The initial pH of BG-11 medium was adjusted to 7.1 before inoculation, according to the medium preparation guidelines. At the end of cultivation, the final pH values across all BG-11 cultures reached similar levels, ranging between 8.9 and 9.2. The Spirulina and Zarrouk based media used for *A. platensis*, and *Nannochloris* sp. were alkaline, with an initial pH of approximately 9.3, which increased throughout the cultivation period reaching values much higher than 10.5.

*A. platensis* (P-limited), *Nannochloris* sp., and mixed culture were cultivated in 4 L working volume photobioreactors illuminated on the one side by 6000 K cool white LED panels with a photon flux density of 100 μmol m^−2^ s^−1^. The photobioreactors were stirred through aeration (filtered air using 0.2 μm filters) and were placed in a temperature-controlled room (28 °C). The remaining species were cultivated in 500 mL Erlenmeyer flasks with a working volume of 400 mL, under constant temperature at 25 °C, and a 12:12 light/dark cycle was applied with a photon flux density of 100 μmol m^−2^ s^−1^, measured at the bottom surface of the flasks using a light meter (LI-250A, LI-COR, Lincoln, NE, USA), provided by 6000 K white LED lightbulbs placed below the cultures. Conical flasks were also stirred with filtered atmospheric air.

### 3.2. Bioassay Tests

At the end of the cultivation period, which corresponded to the end of the exponential phase, as determined by monitoring total suspended solids (TSS) over time, each microalgal culture was directly applied to bioassay tests to assess its biostimulant potential at different concentrations. Tests were performed using both the whole culture and the supernatant obtained after centrifugation at 4500 rpm for 5 min (Hermle Z 366, Hermle Labortechnik GmbH, Wehingen, Germany). The concentration of microalgal biomass was characterized in terms of TSS after drying at 105 °C, whereas volatile solids (VS) of whole cultures and supernatants were determined after incineration at 550 °C according to Standard Methods [67]. These values were used to prepare the concentrations applied in each bioassay test.

#### 3.2.1. Germination Index

Gibberellin-like effect was determined by assessing the germination index (GI) as described in [4], with some modifications. Specifically, cucumber (*Cucumis sativus*) seeds were used instead of garden cress seeds. Cucumber seeds were selected for the GI assay due to their high sensitivity and reproducibility in detecting phytotoxic and biostimulant effects [13,68]. Experiments were conducted in Petri dishes covered with two filter papers, each containing 15 seeds. Each experiment was conducted in triplicate. A volume of 5 mL of whole culture and supernatant was added, with a biomass concentration of 0.2 and 0.5 g L^−1^ according to literature [4]. Seeds were then incubated at 25 °C in the dark for six days. Deionized water was also prepared for comparison (negative control). GI was calculated according to Equation (1):

(1) $$GI(\%)=\frac{G\cdot L}{G_{w}\cdot L_{w}}\cdot 100$$

where G corresponds to the number of germinated seeds and L to their length, while G_w_ and L_w_ refer to the negative control.

#### 3.2.2. Mung Bean Rooting Bioassay

Auxin-like effect was evaluated through the mung bean (*Vigna radiata*) rooting bioassay as described in [13]. Mung bean seeds were planted and grown for 10 days before seedlings were cut 3 cm below the cotyledon and transferred to glass tubes containing 10 mL of the tested samples. Three concentrations (0.003, 0.03, and 0.3 gVS L^−1^) were applied for the whole cultures and supernatants. The highest concentration was selected based on the lowest VS concentration measured among the tested samples, ensuring that all cultures and supernatants could be evaluated at the same maximum organic loading. Two additional concentrations, one and two orders of magnitude lower, were also tested to cover a broad screening range and to detect possible concentration-dependent stimulatory or inhibitory responses. This approach was adopted to normalize the biostimulant dosage based on the organic fraction of the samples, since the auxin-like activity is attributed to organic bioactive compounds. Utilizing volatile solids (VS) ensures that the observed effects are correlated with the active organic matter, excluding the inert inorganic content. Each condition was performed in triplicate. Samples were incubated at 25 °C under a natural light/dark cycle for fifteen days. After the incubation period, the number of roots were counted, and results are presented compared to the deionized water (negative control).

#### 3.2.3. Cucumber Cotyledon Expansion and Rooting Bioassays

Cucumber (*Cucumis sativus*) cotyledon bioassays were performed according to [34,35], with some modifications, to evaluate both rooting (auxin-like effect) and expansion (cytokinin-like effect). After five days of seed growth in agar in the dark, cotyledons with a small hypocotyl segment (1–2 mm) were excised, weighted and transferred to Petri dishes covered with filter paper. Each Petri dish contained five cotyledons and was supplemented with 6 mL of either whole culture or supernatant with a concentration of 0.5 g L^−1^ based on literature [4]. All treatments were performed in duplicate. Petri dishes were incubated at 25 °C in the dark for five days. At the end of the incubation period, the number of roots formed at the base of each cotyledon was recorded and the weight was measured again to assess expansion. The use of a common endpoint allowed simultaneous evaluation of rooting and cotyledon growth responses under the same conditions.

### 3.3. Biomass Composition Analysis

At the end of cultivation, the microalgal biomass was collected by centrifugation at 4500 rpm for 5 min, washed with deionized water (or 0.5 M NH_4_HCO_3_ for *A. platensis*), and lyophilized (LyoQuest, Telstar, Barcelona, Spain). Freeze-dried biomass was analyzed to determine its nitrogen, phosphorus, and potassium content. Nitrogen content was determined using an elemental analyzer (Eurovector EA3100 Series, Pavia, Italy). Briefly, 0.5–1.5 mg of dried biomass was weighed using a high-precision balance (±0.01 mg) and encapsulated in tin capsules. Samples were introduced into an oxidation/reduction reactor operated at 980 °C under a controlled oxygen supply, ensuring complete oxidation. The resulting gases were passed through a reduction column, separated chromatographically, and quantified using a thermal conductivity detector. Total phosphorus in biomass was determined following persulfate digestion of samples and subsequent quantification by the ascorbic acid colorimetric method according to Standard Methods [67]. For potassium determination, samples were subjected to acid digestion with a mixture of nitric acid (HNO_3_) and hydrogen peroxide (H_2_O_2_) in a volumetric ratio of 1:4 and heated at 100 °C for 2 h. Potassium concentration was quantified using a flame photometer (Corning 410, Sherwood Scientific, Cambridge, UK) based on the principle of atomic emission spectroscopy. In this technique, potassium ions introduced into the flame are thermally excited and emit radiation at a characteristic wavelength (768 nm), with the emitted intensity being proportional to the analyte concentration.

### 3.4. Statistical Analysis

Experimental data for germination index and mung bean rooting bioassays were examined for the effect of microalgal species, concentration, fraction and interactions through three-way ANOVA. Two-way ANOVA was also conducted for the cucumber cotyledon bioassays to evaluate the effects of microalgal species, fraction, and their interaction. One-way ANOVA and Tukey’s test was also performed to examine the statistical differences in the applied treatments for grouping. The normality and homogeneity assumptions were assessed using the Shapiro–Wilk and Levene tests, respectively. For all statistical tests, significance was established at *p* < 0.05 (null hypothesis). The analysis was performed via Minitab 18 software.

## 4. Conclusions

Concerns about the environmental impact of widespread chemical fertilizer use have created an urgent need for sustainable agricultural production systems. In this study, eight photosynthetic cultures were screened to evaluate their potential as biostimulants and biofertilizers through a combination of bioassays and nutrient composition analysis. The results demonstrated that biostimulant responses are strongly dependent on concentration, sample fraction, and species, highlighting the complexity of the process. Under the cultivation and application conditions tested, and considering the composite score presented in Table 3 as a preliminary prioritization tool rather than as a validated framework for definitive candidate selection, *C. vulgaris*, mixed culture, and *A. obliquus* emerged as promising candidates for further evaluation as biostimulants and biofertilizers. However, the findings indicate that microalgal biomass cannot be considered as a uniform input, since its effectiveness depends on parameters that should be carefully selected based on the species and the intended agronomic application. Further investigation is required to optimize cultivation conditions, as nutrient availability and cultivation conditions can affect both biomass composition and bioactivity. Despite the promising potential of microalgal biofertilizers, their large-scale application remains challenging due to scalability, production cost, and downstream processing challenges. Harvesting and biomass separation can become economically demanding because of high energy and maintenance requirements, while drying methods are constrained by weather dependence, long processing times, large area requirements, or high operating costs. Further work should therefore assess low-cost production systems, product stability, technoeconomic feasibility, and the risks of repeated field application [7]. Alternative low-cost substrates should be investigated to enhance the environmental sustainability and economic feasibility of the process. In this context, species capable of tolerating saline or marine conditions could represent particularly attractive candidates, as their cultivation in seawater or brackish water may reduce freshwater demand and overall production costs. This potential benefit should be further investigated through dedicated saline or brackish cultivation experiments. In conclusion, the present laboratory-scale findings provide preliminary evidence that microalgae may contribute to the development of alternative agricultural inputs. However, their agronomic effectiveness under realistic agricultural conditions remains to be confirmed through greenhouse and field-scale validation trials, including assessment across different crops, soils, application rates, and environmental conditions.

## Figures and Tables

**Table 1:** Nitrogen, phosphorus, and potassium content (% *w*/*w*) in microalgal biomass. Data are presented as mean values with their standard deviation (n = 3).

Species	N [% *w*/*w*]	P [% *w*/*w*]	K [% *w*/*w*]
*A. platensis* (P-limited)	4.3 ± 0.1 ^e^	0.3 ± 0.1 ^e^	0.7 ± 0.0 ^c^
*Nannochloris* sp.	6.2 ± 0.3 ^d^	1.0 ± 0.1 ^d,e^	0.9 ± 0.3 ^c^
*Mixed culture*	6.5 ± 0.2 ^c,d^	4.6 ± 0.5 ^a^	0.0 ± 0.0 ^d^
*A. platensis*	6.9 ± 0.2 ^b,c^	1.4 ± 0.3 ^c,d^	2.1 ± 0.3 ^a^
*C. vulgaris*	8.2 ± 0.4 ^a^	3.0 ± 0.8 ^b^	1.3 ± 0.2 ^b^
*Chlorella* sp.	8.8 ± 0.1 ^a^	1.2 ± 0.2 ^d,e^	0.7 ± 0.1 ^c^
*A. obliquus*	8.4 ± 0.3 ^a^	1.7 ± 0.4 ^c,d^	0.8 ± 0.0 ^c^
*P. kessleri*	7.4 ± 0.1 ^b^	2.5 ± 0.3 ^b,c^	0.8 ± 0.0 ^c^
*C. vacuolata*	6.6 ± 0.1 ^c,d^	1.9 ± 0.0 ^b,c,d^	1.0 ± 0.0 ^b,c^

^a,b,c,d,e^ Data in the same column that do not share a letter are significantly different based on Tukey test, *p* < 0.05.

**Table 2:** Microalgae species applications in agriculture and their potential use as biofertilizers.

Species	Cultivation Medium	N, P, K [%] Content in Biomass	Crop	Effect	Reference
Mixed biomass dominated by *Scenedesmus* from domestic wastewater	Domestic wastewater	7.6, 1.6, 0.9	*Ocimum* *basilicum* L.	Leaf dry weight, was 27–28% higher in microalgae treatment	[57]
*Limnospira* sp.	Brewery wastewater	4.0, 0.6, 0.6	*Hordeum* *vulgare*	Increased productivity, protein content, and grain size by 26.9%, 14.4%, and 8.78%,	[58]
Algal consortium (*Chlorella* sp. and *Scenedesmus* sp.)	Domestic wastewater	7.8, 1.7, 1.0	*Solanum* *lycopersicum*	50% microalgal biomass increased 32% tomato yield	[59]
*C. minutissima*	Sewage wastewater	6.0, 1.0, 0.5	Spinach and baby corn	Both crops showed equivalent or superior performance when using 100% algal biomass compared to conventional mineral	[60]
*Tetraselmis* sp.	Municipal wastewater	6.9, 0.8, 1.3	Wheat	Increased plant height of up to 13% and number of leaves of up to 50% compared to chemical fertilizer	[61]
*Nannochloropsis* sp.	Municipal wastewater	7.4, 0.4, 1.2	Wheat	Increased plant height of up to 23%, number of leaves of up to 25% and leaf length of up to 27% compared to chemical fertilizer	[61]
*Chlorella* sp.	Municipal wastewater	4.8, 0.3, 0.7	Wheat	Increased number of leaves of up to 125% and leaf length of up to 35% compared to chemical fertilizer	[61]
*Scenedesmus* sp.	Municipal wastewater	5.0, 0.4, 0.8	Wheat	Increased plant height of up to 24% and number of leaves of up to 150% compared to chemical fertilizer	[61]

**Table 3:** Exploratory comparative scoring of the tested microalgal cultures based on bioassay responses and nutrient content.

Species	GI	Mung Bean Rooting	Cucumber Rooting	Cucumber Expansion	N	P	K	Final Score
*A. platensis* (P-limited)	0.88	0.86	0.29	0.54	0.00	0.00	0.33	2.90
*Nannochloris* sp.	0.59	0.64	0.14	0.58	0.42	0.16	0.43	2.95
*Mixed culture*	1.00	0.86	0.84	0.94	0.49	1.00	0.00	5.12
*A. platensis*	0.54	0.86	0.08	0.58	0.58	0.25	1.00	3.88
*C. vulgaris*	0.97	0.86	1.00	0.70	0.87	0.62	0.62	5.63
*Chlorella* sp.	0.94	1.00	0.90	0.47	1.00	0.21	0.33	4.85
*A. obliquus*	0.98	0.86	0.61	0.84	0.91	0.32	0.38	4.90
*P. kessleri*	0.89	0.80	0.63	0.71	0.69	0.50	0.38	4.61
*C. vacuolata*	0.88	0.79	0.65	1.00	0.51	0.37	0.48	4.68

## Data Availability

Data will be made available upon request.

## Supplementary Materials

The following supporting information can be downloaded at https://www.mdpi.com/article/10.3390/md24070228/s1, Table S1. Three-way ANOVA testing the effect of the microalgal species, concentration, fraction, and their interactions on the germination index of cucumber. The data variability is attributable to the main effect of each factor and the interaction found, as indicated by the *p*-value. The contribution of each factor was expressed as the percentage variation in the response (*F*-ratio of each factor relative to the sum of all *F*-ratios), Table S2. Grouping information using the Tukey method and 95% confidence for the germination index of each treatment, Table S3. Three-way ANOVA testing the effect of the microalgal species, concentration, fraction, and their interaction on the mung bean rooting bioassay. The data variability is attributable to the main effect of each factor and the interaction found, as indicated by the *p*-value. The contribution of each factor was expressed as the percentage variation in the response (*F*-ratio of each factor relative to the sum of all *F*-ratios), Table S4. Two-way ANOVA testing the effect of the microalgal species, fraction, and their interaction on the cucumber cotyledon rooting and expansion. The data variability is attributable to the main effect of each factor and the interaction found, as indicated by the *p*-value. The contribution of each factor was expressed as the percentage variation in the response (*F*-ratio of each factor relative to the sum of all *F*-ratios).

## Abbreviations

The following abbreviations are used in this manuscript:

GI	Germination Index
TSS	Total Suspended Solids
VS	Volatile Solids
