# Supplementary material for: Screening of Microalgal Species for Biostimulant and Biofertilizer Applications
Source: Mar Drugs. 2026 Jun 29;24(7):228. doi: 10.3390/md24070228 (PMC13412809; doi:10.3390/md24070228)
Supplement: Supplementary file 1 [file marinedrugs-24-00228-s001.zip › marinedrugs-4332517-supplementary.pdf]

Article

Screening of Microalgal Species for Biostimulant and Biofertilizer Applications

Eirini Sventzouri [1], Eleni Pagkaki [1], Sotirios Zerveas [2], Giorgos Markou [2] and Michael Kornaros [1,*]

[1] Laboratory of Biochemical Engineering & Environmental Technology (LBEET), Department of Chemical Engineering, University of Patras, 26504 Patras, Greece; eirinisventzouri@gmail.com (E.S.); helenpag@hotmail.com (E.P.)
[2] Institute of Technology of Agricultural Products, Hellenic Agricultural Organization—DIMITRA, Sof. Venizelou 1, 14123 Lykovrysi, Greece; sotzerveas@gmail.com (S.Z.); markougior@elgo.gr (G.M.)
* Correspondence: kornaros@chemeng.upatras.gr

Table S1. Three-way ANOVA testing the effect of the microalgal species, concentration, fraction, and their interactions on the germination index of cucumber. The data variability is attributable to the main effect of each factor and the interaction found, as indicated by the *p*-value. The contribution of each factor was expressed as the percentage variation of the response (*F*-ratio of each factor relative to the sum of all *F*-ratios).

Species	Variation [%]	p-value
Species	27.31	<0.001
Concentration	48.92	<0.001
Fraction	3.71	0.001
2-factors interaction		
Species × Concentration	13.52	<0.001
Species × Fraction	2.36	<0.001
Concentration × Fraction	2.32	0.007
3-factors interaction		
Species × Concentration × Fraction	1.86	<0.001

Table S2. Grouping information using the Tukey method and 95% confidence for the germination index of each treatment.

Treatment	Grouping
Mixed culture 0.2 g L^{-1} whole culture	a
A. obliquus 0.5 g L^{-1} supernatant	a
A. obliquus 0.5 g L^{-1} whole culture	a,b
C. vulgaris 0.5 g L^{-1} whole culture	a,b
Mixed culture 0.2 g L^{-1} supernatant	a,b
Chlorella sp. 0.5 g L^{-1} whole culture	a,b
C. vulgaris 0.2 g L^{-1} whole culture	a,b
Chlorella sp. 0.5 g L^{-1} supernatant	a,b
A. obliquus 0.2 g L^{-1} supernatant	a,b
C. vulgaris 0.2 g L^{-1} supernatant	a,b
Mixed culture 0.5 g L^{-1} supernatant	a,b
Chlorella sp. 0.2 g L^{-1} whole culture	a,b
P. kessleri 0.2 g L^{-1} whole culture	a,b,c
A. platensis (P-limited) 0.2 g L^{-1} whole culture	a,b,c
C. vacuolata 0.5 g L^{-1} whole culture	a,b,c
C. vulgaris 0.5 g L^{-1} supernatant	a,b,c
Mixed culture 0.5 g L^{-1} whole culture	a,b,c,d
C. vacuolata 0.5 g L^{-1} supernatant	a,b,c,d,e
Chlorella sp. 0.2 g L^{-1} supernatant	a,b,c,d,e
A. platensis (P-limited) 0.2 g L^{-1} supernatant	a,b,c,d,e
P. kessleri 0.2 g L^{-1} supernatant	a,b,c,d,e
P. kessleri 0.5 g L^{-1} whole culture	a,b,c,d,e
C. vacuolata 0.2 g L^{-1} whole culture	a,b,c,d,e,**f**
C. vacuolata 0.2 g L^{-1} supernatant	a,b,c,d,e,**f**
A. obliquus 0.2 g L^{-1} whole culture	b,c,d,e,**f**
P. kessleri 0.5 g L^{-1} supernatant	b,c,d,e,**f**
Nannochloris sp. 0.2 g L^{-1} supernatant	c,d,e,**f**
Nannochloris sp. 0.2 g L^{-1} whole culture	d,e,**f**
A. platensis 0.2 g L^{-1} whole culture	e,**f**
A. platensis 0.2 g L^{-1} supernatant	**f**
A. platensis (P-limited) 0.5 g L^{-1} supernatant	g
A. platensis 0.5 g L^{-1} supernatant	g
A. platensis 0.5 g L^{-1} whole culture	g
Nannochloris sp. 0.5 g L^{-1} whole culture	g
Nannochloris sp. 0.5 g L^{-1} supernatant	g

[a,b,c,d,e,f,g] Data that do not share a letter are significantly different based on Tukey test, $p<0.05$.

Table S3. Three-way ANOVA testing the effect of the microalgal species, concentration, fraction, and their interaction on the mung bean rooting bioassay. The data variability is attributable to the main effect of each factor and the interaction found, as indicated by the *p*-value. The contribution of each factor was expressed as the percentage variation of the response (*F*-ratio of each factor relative to the sum of all *F*-ratios).

**Species**	**Variation [%]**	**p-value**
Species	6.12	<0.001
Concentration	35.77	<0.001
Fraction	48.93	<0.001
2-factors interaction		
Species × Concentration	1.86	<0.001
Species × Fraction	2.17	<0.001
Concentration × Fraction	3.01	<0.001
3-factors interaction		
Species × Concentration × Fraction	2.14	<0.001

Table S4. Two-way ANOVA testing the effect of the microalgal species, fraction and their interaction on the cucumber cotyledon rooting and expansion. The data variability is attributable to the main effect of each factor and the interaction found, as indicated by the *p*-value. The contribution of each factor was expressed as the percentage variation of the response (*F*-ratio of each factor relative to the sum of all *F*-ratios).

**Species**	**Variation [%]**		**p-value**	
	Rooting	Expansion	Rooting	Expansion
Species	25.67	17.21	<0.001	<0.001
Fraction	68.26	66.42	<0.001	<0.001
2-factors interaction				
Species × Fraction	6.07	16.37	0.021	<0.001
